# Computed Tomography Findings as Determinants of Local and Systemic Inflammation Biomarkers in Interstitial Lung Diseases: A Retrospective Registry-Based Descriptive Study

**DOI:** 10.1007/s00408-021-00434-w

**Published:** 2021-03-26

**Authors:** David Lang, Kaveh Akbari, Andreas Horner, Magdalena Hepp, Bernhard Kaiser, Herwig Pieringer, Bernd Lamprecht

**Affiliations:** 1 Department of Pulmonology, Kepler University Hospital GmbH, Krankenhausstrasse 9, 4020 Linz, Austria; 2 Central Radiology Institute, Kepler University Hospital GmbH, Krankenhausstrasse 9, 4020 Linz, Austria; 3 Diakonissen Hospital Linz, Weissenwolfstrasse 15, 4020 Linz, Austria; 4 Institute of General, Family and Preventive Medicine, Paracelsus Medical University, Strubergasse 21, 5020 Salzburg, Austria

**Keywords:** Broncho-alveolar lavage, Peripheral blood, C-reactive protein, Eosinophil, Lymphocyte, Idiopathic pulmonary fibrosis, Chronic hypersensitivity pneumonitis

## Abstract

**Purpose:**

To evaluate the association of peripheral blood (PBL) and broncho-alveolar lavage (BAL) biomarkers with inflammatory versus fibrotic high-resolution computed tomography (HRCT) findings in interstitial lung disease (ILD) patients.

**Methods:**

HRCT findings of 127 consecutive ILD-board patients were semi-quantitatively evaluated: reticulation/honeycombing (RET), traction bronchiectasis (TBR) and emphysema (EMP) were classified as non-inflammatory/fibrotic; consolidations (CON), ground glass opacities (GGO), parenchymal nodules (NDL) and mosaic attenuation (MOS) as active inflammatory. Each HRCT finding was assessed in six distinct lung regions, resulting scores were graded as minimal (0–1 regions involved), medium (2–4) or extensive (5–6). Associations of routinely assessed PBL/BAL biomarkers with these HRCT scores were evaluated using Spearman correlation coefficients and graphical presentation; significance was tested by applying Kruskal–Wallis tests.

**Results:**

Blood neutrophil, lymphocyte and eosinophil fraction, neutrophil to lymphocyte ratio (NLR) and BAL lymphocyte fraction consistently showed opposite correlations with inflammatory versus non-inflammatory/fibrotic HRCT finding scores. Blood lymphocyte fraction significantly differed by graded GGO (*p* = 0.032) and CON (*p* = 0.027) extent, eosinophil fraction by TBR (*p* = 0.006) and NLR by CON (*p* = 0.009). C-reactive protein was significantly related to GGO (*p* = 0.023) and CON (*p* = 0.004), BAL lymphocyte fraction to GGO (*p* = 0.017) extent.

**Conclusion:**

Blood lymphocyte and eosinophil fraction, NLR, CRP and BAL lymphocyte fraction may aid to differentiate inflammatory from non-inflammatory/fibrotic ILD patterns.

**Trial registration:**

This evaluation was based on data from the ILD registry of Kepler University Hospital Linz, as approved by the ethics committee of the Federal State of Upper-Austria (EK Number. I-26-17).

**Supplementary Information:**

The online version contains supplementary material available at 10.1007/s00408-021-00434-w.

## Introduction

The anti-fibrotic drugs pirfenidone and nintedanib decelerate lung function decline in idiopathic pulmonary fibrosis (IPF) and progressive fibrosing interstitial lung diseases (PFILD) [1–4], while patients with ILD susceptible to immunomodulatory therapies can experience an improvement in both radiological imaging and pulmonary function tests [5–7]. Still, also ILD with an “inflammatory” origin like systemic sclerosis associated ILD (SSCILD) or chronic hypersensitivity pneumonitis (CHP) frequently present with a PFILD phenotype [8, 9]. A combination of prednisone, azathioprine and N-acetylcysteine led to adverse outcomes in IPF patients [10, 11], while in SSCILD placebo-controlled trials have provided evidence on the efficacy of immunosuppressive as well as anti-fibrotic therapies [6, 12, 13]. In various fibrotic ILD apart from IPF it is still unclear, whether an anti-fibrotic, an immunosuppressive or a combined approach is most beneficial.

In current clinical practice in ILD, most biomarker information is derived from high-resolution computed tomography (HRCT) imaging. A radiological pattern of usual interstitial pneumonia (UIP) irrespective of the underlying etiology bears a poorer prognosis than possible UIP or nonspecific interstitial pneumonia (NSIP) [14–20]. Under certain conditions, an UIP-pattern can be diagnostic for IPF and lead to the initiation of anti-fibrotic therapy without the need of lung biopsy [19, 20]. In most ILD cases, however, HRCT patterns are not uniform but rather involve several coexisting abnormalities for example reticulation (RET), ground glass opacities (GGO) and traction bronchiectasis (TBR) in fibrotic NSIP. The relative distribution and extent of such radiological findings depends on the underlying pathogenetic processes, the course and duration of disease [15].

Hypothetically, peripheral blood (PBL) and broncho-alveolar lavage (BAL) biomarkers could help to differentiate ILD cases with an inflammatory from those with a predominantly fibrotic phenotype. Knowledge on their interaction with HRCT findings could aid the development of biomarkers guiding ILD therapy in the future.

## Methods

Based on a retrospective ILD registry cohort, we have evaluated routinely assessed biomarkers from PBL and BAL fluid for their association with a set of visually semi-quantified HRCT finding scores.

This study was performed according to the Strengthening the Reporting of Observational Studies in Epidemiology (STROBE) guidelines for reporting observational studies [21]. Patient data used for this analysis were retrieved from the ILD registry of Kepler University Hospital Linz, Austria. The registry as well as the present evaluation have been conducted in concordance with the Declaration of Helsinki and were approved and re-assessed on a yearly basis by the ethics committee of the Federal State of Upper Austria (Study number I-26-17). All patients enrolled were subsequently discussed by the monthly local ILD-board after they had undergone a standardized ILD evaluation program including a structured assessment of patient history and physical examination, HRCT imaging, pulmonary function tests and laboratory analyses with standard autoimmune serologies [20, 22]. Patients in whom ILD board discussion resulted in no ILD diagnosis were excluded from this study.

HRCT images were acquired according to protocols suggested by the relevant guidelines [19, 20, 23]. If clinically feasible, prone imaging was preferred to differ opacities in dependent lung areas from true interstitial lung abnormalities [24].

Blood samples were analyzed using a Sysmex® XN-3000 hematology analyzer (Sysmex Europe GmbH, Norderstedt, Germany) for blood cell counts and a Cobas® 8000 modular analyzer (Roche Diagnostics International AG, Rotkreuz, Switzerland) for C-reactive protein (CRP) and lactate dehydrogenase (LDH).

Broncho-alveolar lavage was performed according to the relevant guidelines [25, 26], when clinically indicated by the treating physician or by the ILD-board. A total of 100 mL of 0.9% saline was instilled and retrieved in aliquots of 20 mL via flexible bronchoscopy under sedoanalgesia. The BAL location was a segmental bronchus of either one of the upper lobes including the lingula or the middle lobe at the discretion of the conducting physician according to the location of most active or extensive disease in HRCT. BAL samples were prepared using 100 µL of BAL fluid on a Tharmac® Cellspin I cytocentrifuge (Tharmac GmbH, Wiesbaden, Germany) at 700 rounds per minute for 5 min and Wright Giemsa staining. Cell counting was performed manually under 400-fold magnification, cell fractions were given as % of the total cell count, excluding epithelial cells or erythrocytes. Further lymphocyte subset analyses were performed in BAL samples with a lymphocyte fraction of ≥ 15%.

To allow for statistical analyses of HRCT scans, we have previously devised a semi-quantitative scoring system based on four elementary lesion types: parenchymal nodules (NDL), reticular abnormalities (interlobular septal and intralobular interstitial thickening and honeycombing—RET), increased lung attenuation (consolidations (CON), ground glass opacities (GGO)) and reduced lung attenuation (emphysema—EMP) findings. Besides, the extent of mosaic attenuation (mosaic perfusion, air-trapping—MOS) and traction bronchi(-ol)ectasis (TBR) were assessed. [23, 27, 28]. For quantification, both lungs were separated in an upper-, middle- and lower-lung area as defined by thirds of the largest cranio-caudal diameter in the sagittal reconstructions, leading to six distinct lung areas. The individual extent of each quantified HRCT pattern (RET, TBR, EMP, CON, GGO, NDL, MOS) was calculated as the sum of all involved defined lung areas (0–6). The described HRCT scoring process was accomplished during the respective ILD-board session by a specialized ILD radiologist in a non-blinded fashion.

To evaluate the associations between PBL and BAL inflammation biomarkers and the quantified imaging features, correlation coefficients were calculated for each HRCT finding score and each PBL and BAL biomarker. Direction, strength, and significance of these correlations were depicted in color-coded tables for visual analysis. To test for clinically relevant significance of these associations, groups with no or minimal (0–1), medium (2–4) and extensive HRCT involvement (5–6) were compared using the Kruskal–Wallis test. All statistical analyses were performed using R (R: A Language and Environment for Statistical Computing; Version 3.6.0; https://www.R-project.org). For all tests performed, a *p*-value < 0.05 was regarded statistically significant.

## Results

We evaluated 127 ILD patients consecutively discussed by the multidisciplinary ILD-board of Kepler University Hospital Linz, Austria between February 2017, and September 2018. Clinical and radiological patient characteristics are shown in Tables 1 and 2. The fraction of patients with “other ILD” (*n* = 23; 18%) included nine cases of organizing pneumonia (OP), six patients with sarcoidosis, three with respiratory-bronchiolitis-ILD, two with drug-associated pneumonitis and one patient each with pulmonary Langerhans-cell histiocytosis, pleuro-parenchymal fibroelastosis and lymphangioleiomyomatosis. Eleven patients (9%) were considered “unclassified ILD”, either due to patients not willing to undergo further necessary diagnostic steps like lung biopsy or to situations, where further work-up was deemed inappropriate due to age or major comorbidities. Eleven patients (9%; ten with NSIP, one with unclassifiable ILD) were or had already been diagnosed with autoimmune disorders considered causally related to ILD (rheumatoid arthritis in four patients, autoimmune-hepatitis in two patients, Sjögren’s syndrome in two patients and pauci-immune glomerulonephritis, granulomatosis with polyangiitis and SHARP-syndrome in one patient each).

**Table 1:** Patient characteristics

Patient characteristics		Comorbidities	*n* (%)
Mean age (years, SD)	65 (14)	None	17 (13)
Age range (years)	18–91	Pulmonary	
Male sex (*n*, %)	82 (65)	Chronic obstructive pulmonary disease	15 (12)
Reported onset of respiratory symptoms (years, SD)	4.1 (5.8)	Asthma	8 (6)
Family history of ILD (*n*, %)	8 (6)	History of tuberculosis	7 (6)
		Cardiovascular	
		Arterial hypertension	46 (36)
ILD-board diagnosis	*n* (%)	Coronary artery disease	22 (17)
		Atrial fibrillation	19 (15)
Interstitial pneumonia with autoimmune features	26 (20)	Chronic kidney disease	8 (6)
Idiopathic pulmonary fibrosis	23 (18)	Stroke/cerebrovascular disease	6 (5)
Idiopathic non-specific interstitial pneumonia	16 (13)	Peripheral artery disease	5 (4)
Associated ILD	11 (9)	Endocrine	
Unclassified ILD	11 (9)	Diabetes mellitus	19 (15)
Other ILD	23 (18)	Thyroid disorder	13 (10)
		Hyperlipidemia	10 (8)
		Osteoporosis	5 (4)
Smoking history	*n* (%)	Gastrointestinal	
Mean pack years (mean, SD)	19.4 (25.2)	Gastroesophageal reflux disease	17 (13)
Never smoker	52 (41)	Viral hepatitis	7 (6)
Former smoker	50 (39)	Autoimmune	
Current smoker	17 (13)	Related to ILD*	11 (9)
Exclusively passive smoker	6 (5)	Psoriasis	7 (6)
		Malignancy (solid and haematological)	12 (9)

Data are given as *n* (%) unless otherwise specified. Only comorbidities present in five or more patients are listed

*Specified in the results section

*SD* Standard deviation, *ILD* Interstitial lung disease

**Table 2:** Peripheral blood, broncho-alveolar lavage, and HRCT characteristics

Peripheral blood biomarkers	*n* (%)	Mean (SD)
Leukocyte count (G/L)	122 (96)	8.7 (3.4)
Neutrophil fraction (%)	121 (95)	70.9 (10.9)
Lymphocyte fraction (%)		20.5 (8.7)
Neutrophil to lymphocyte ratio		5 (4.9)
Eosinophil fraction (%)		0.2 (0.3)
C-reactive protein (mg/dL)	123 (97)	1.6 (2.6)
Lactate dehydrogenase (U/L)	113 (89)	247 (78.2)

BAL biomarkers	*n* (%)	mean (SD)
Macrophage fraction (%)	66 (52)	51.9 (29.9)
Neutrophil fraction (%)		18.5 (23.1)
Lymphocyte fraction (%)		18.5 (21.6)
Eosinophil fraction (%)		3.6 (7.9)
CD4 + /CD8 + ratio	20 (16)	2.3 (1.9)

HRCT finding scores	*n* (%)	Median (range)
Parenchymal nodules	40 (31)	0 (0–6)
Reticulation/honeycombing	106 (83)	4 (0–6)
Honeycombing	22 (17)	0 (0–6)
Ground glass opacities	49 (39)	0 (0–6)
Consolidations	44 (35)	0 (0–6)
Emphysema	23 (18)	0 (0–6)
Traction bronchiectasis	100 (79)	2 (0–6)
Mosaic attenuation	32 (25)	0 (0–6)

Values are given as *n* (%) and mean (SD) or median (range) as specified

*SD* Standard deviation, *IQR* Interquartile range, *BAL* Broncho-alveolar lavage, *CD* Cluster of differentiation, *HRCT* High-resolution computed tomography

HRCT, PBL and BAL characteristics according to ILD-board diagnoses are shown in Supplementary Table 1.

**Fig. 1:**
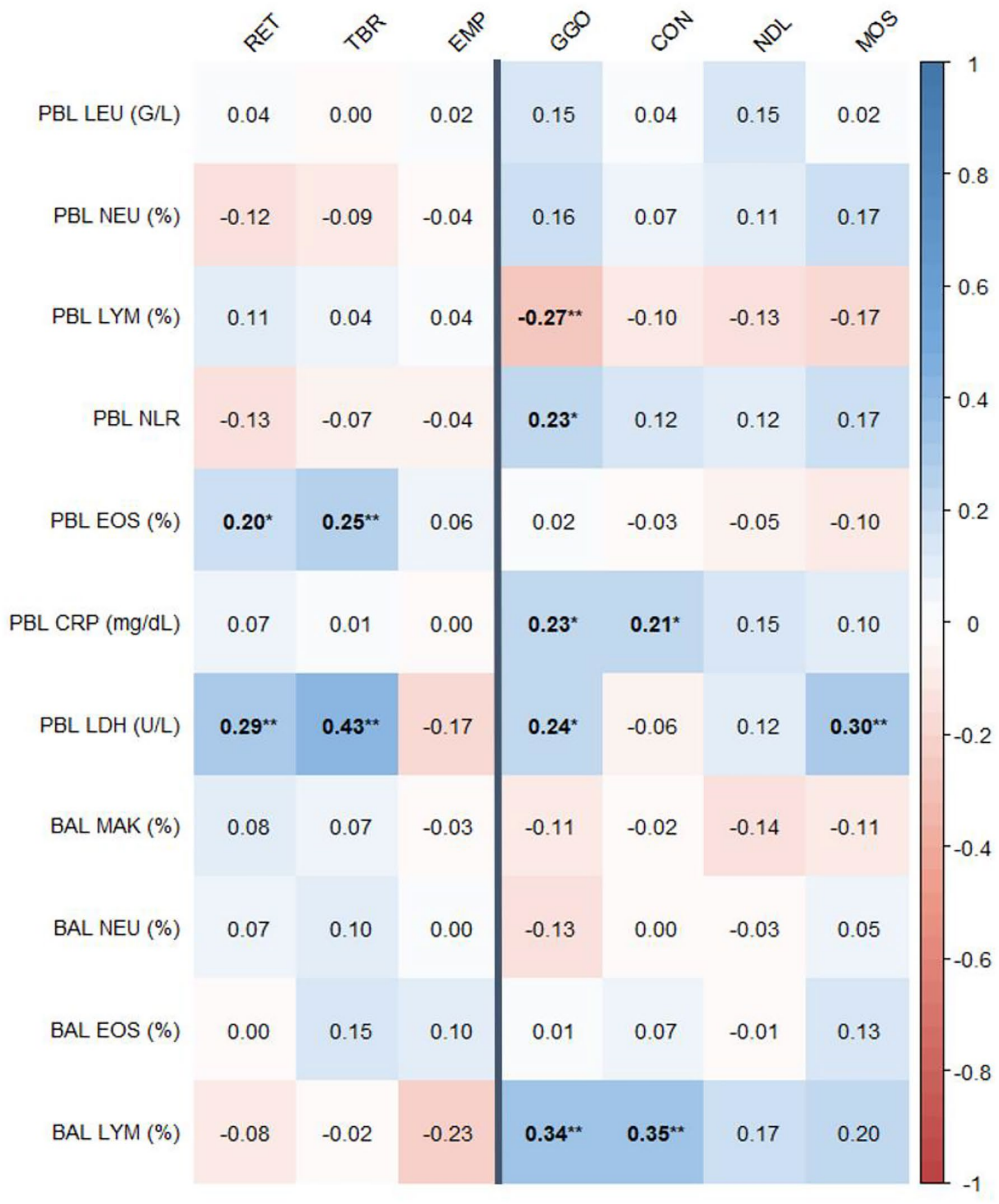
Correlation matrix of peripheral blood and broncho-alveolar lavage biomarkers with HRCT finding scores. Values are for Spearman correlation coefficients; colors indicate strength and direction of correlations as shown by the scale on the right side. Bold numbers are for significant correlations, *p < 0.05, **p < 0.01. The line between the EMP and GGO category visually separates non-inflammatory/fibrotic from inflammatory HRCT findings. *PBL* Peripheral blood, *BAL* Broncho-alveolar lavage, *HRCT* High-resolution computed tomography, *RET* Reticulation/honeycombing, *TBR* Traction bronchiectasis, *EMP* Emphysema, *GGO* Ground glass opacities, *CON* Consolidations, *NDL* Parenchymal nodules, *MOS* Mosaic attenuation, *LEU* Leukocyte count, *NEU* Neutrophil fraction, *LYM* Lymphocyte fraction, *NLR* Neutrophil to lymphocyte ratio, *EOS* Eosinophil fraction, *CRP* C-reactive protein, *LDH* Lactate dehydrogenase, *MAK* Macrophage fraction

Correlations of PBL and BAL biomarkers with HRCT finding scores are shown in Fig. 1. Blood neutrophil, lymphocyte and eosinophil fraction, neutrophil to lymphocyte ratio (NLR) and BAL lymphocyte fraction showed consistently opposite correlations for inflammatory versus non-inflammatory/fibrotic HRCT finding scores. Significant correlations were seen for PBL lymphocyte fraction and GGO (*r* = − 0.27, *p* < 0.01), PBL NLR and GGO (*r* = 0.23, *p* < 0.05) and for PBL eosinophil fraction and RET (*r* = 0.2, *p* < 0.05) as well as TBR (*r* = 0.25, *p* < 0.01). Concerning CRP, GGO (*r* = 0.23, *p* < 0.05) and CON (*r* = 0.21, *p* < 0.05) showed significant interactions, while LDH was significantly correlated to RET (r = 0.29, *p* < 0.01), TBR (*r* = 0.43, p < 0.01), GGO (*r* = 0.24, *p* < 0.05) and MOS (*r* = 0.3, *p* < 0.01). Among BAL biomarkers, only lymphocyte fraction was significantly correlated with HRCT finding scores, namely with GGO (*r* = 0.34, *p* < 0.01) and CON (*r* = 0.35, *p* < 0.01).

**Fig. 2:**
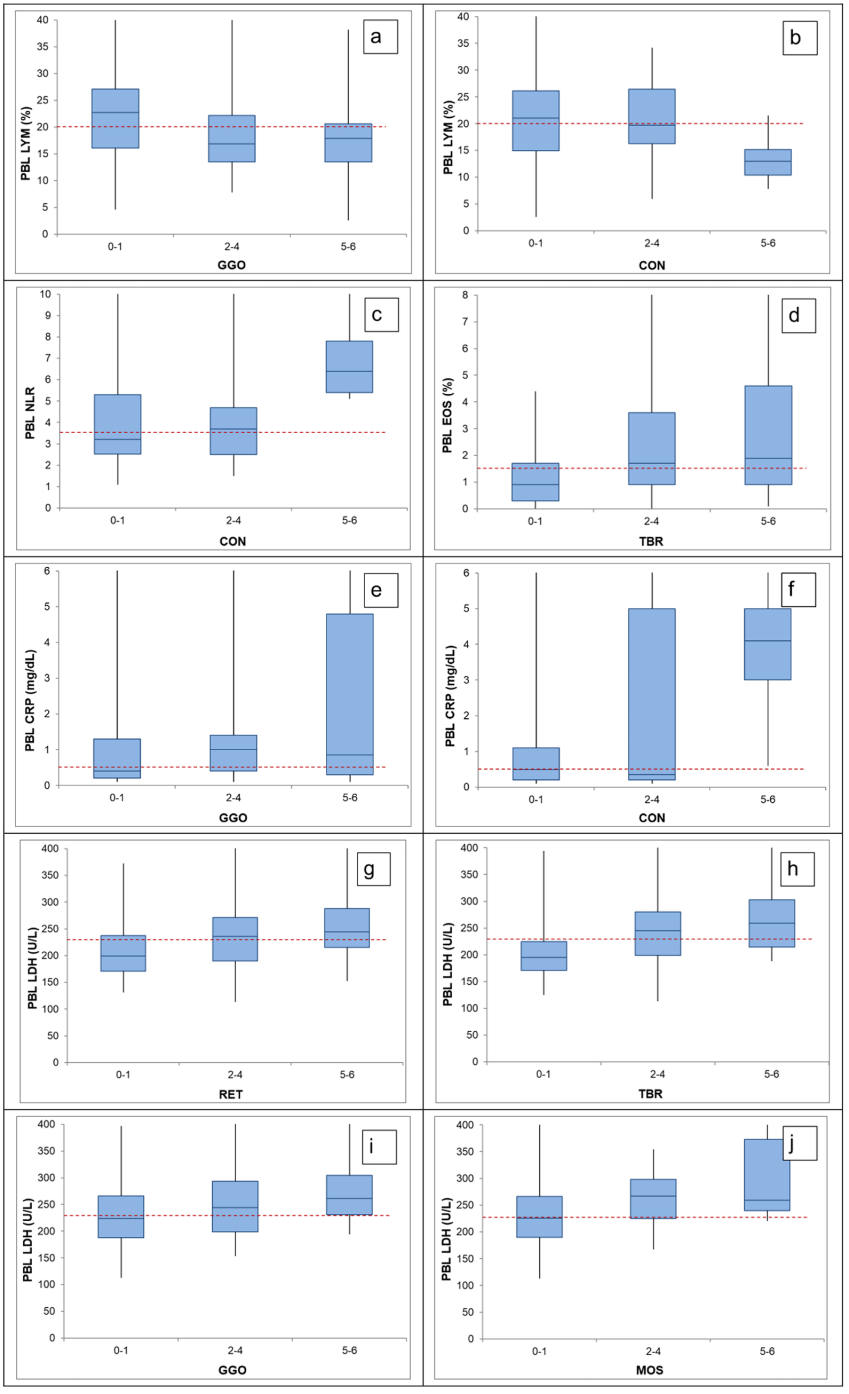
Boxplot diagrams of significant findings for PBL biomarkers according to HRCT score categories. Boxplot diagrams depict the median (line), the upper and lower quartile (boxes) and the 95% confidence intervals (whiskers). The red dashed line represents the median of all values. *PBL* Peripheral blood, *LYM* Lymphocyte fraction, *GGO* Ground glass opacities, *CON* Consolidations, *NLR* Neutrophil to lymphocyte ratio, *EOS* Eosinophil fraction, *TBR* Traction bronchiectasis, *CRP* C-reactive protein, *LDH* Lactate dehydrogenase, *RET* Reticulation/honeycombing, *MOS* Mosaic attenuation, *HRCT* High-resolution computed tomography

**Fig. 3:**
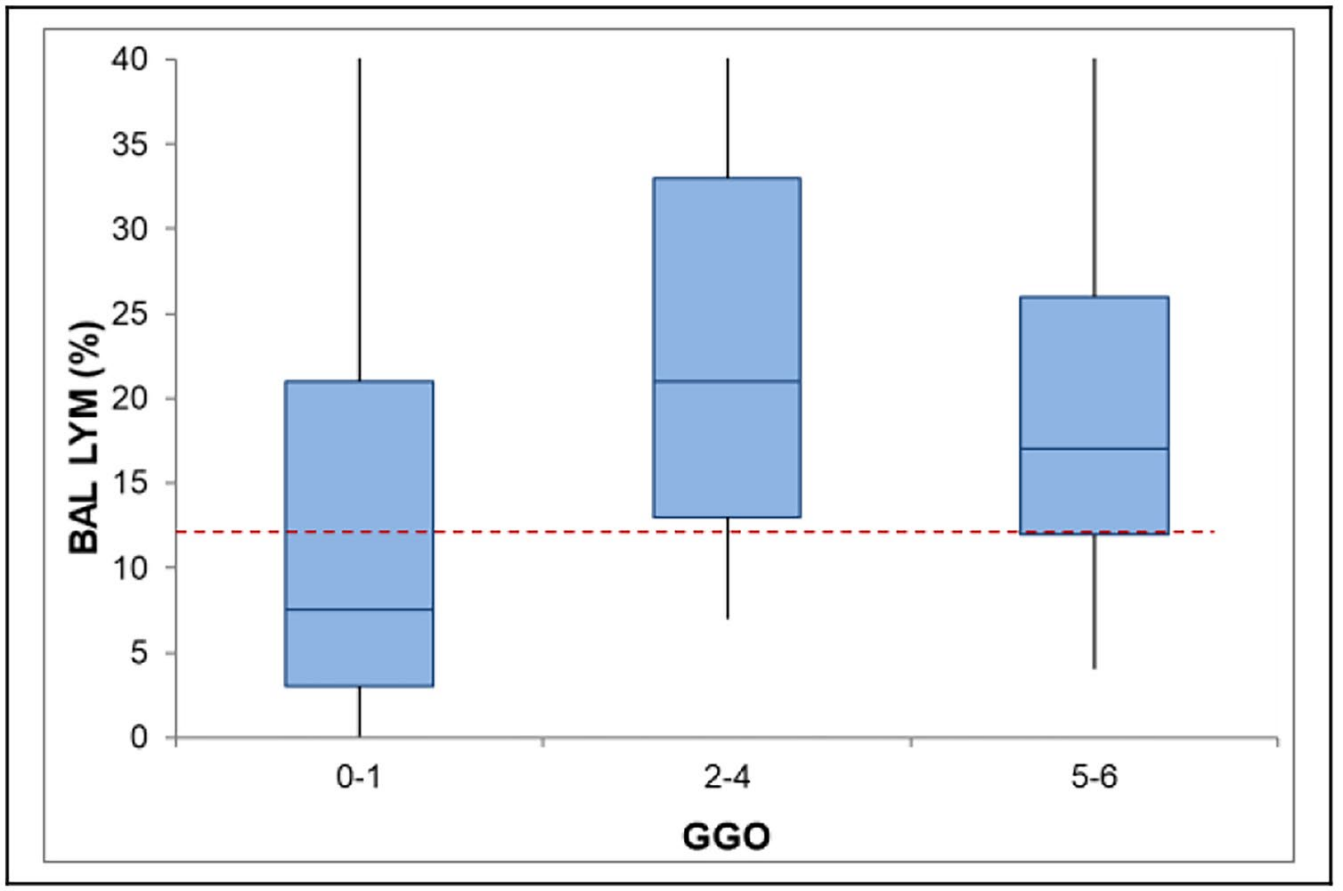
Boxplot diagram of BAL lymphocyte fraction according to GGO score categories. Boxplot diagrams depict the median (line), the upper and lower quartile (boxes) and the 95% confidence intervals (whiskers). The red dashed line represents the median of all values. *BAL* Broncho-alveolar lavage, *LYM* Lymphocyte fraction, *GGO* ground glass opacities

As shown in Figs. 2 and 3 as well as in Supplementary Tables 2 and 3, the categorized scores for the extent of HRCT findings were used to test for clinically meaningful significance of the associations with each PBL or BAL biomarker variable. Blood lymphocyte fraction significantly differed by graded GGO (*p* = 0.032, Fig. 2a) and CON (*p* = 0.027, Fig. 2b), blood NLR by CON (*p* = 0.009, Fig. 2c) and blood eosinophil fraction by TBR (*p* = 0.006, Fig. 2d) extent. CRP was significantly related to GGO (*p* = 0.023, Fig. 2e) and CON (*p* = 0.004, Fig. 2f), while LDH showed significant associations with RET (*p* = 0.01, Fig. 2g), TBR (*p* < 0.001, Fig. 2h), GGO (*p* = 0.049, Fig. 2i) and MOS (*p* = 0,027, Fig. 2j).

Concerning BAL biomarkers, the only significant interaction was shown for BAL lymphocyte fraction and GGO (*p* = 0.017, Fig. 3). Analyses on BAL cluster of differentiation (CD)4 + /CD8 + ratio were only available in a minority of patients and revealed close association with the extent of parenchymal nodules (Supplementary Figure 1 and Supplementary Table 4).

## Discussion

In summary, our analyses indicate that PBL lymphocyte and eosinophil fraction, neutrophil to lymphocyte ratio (NLR), CRP as well as BAL lymphocyte fraction may have clinically relevant implications in differing HRCT abnormalities indicating either active inflammation (ground glass opacities, consolidations, parenchymal nodules, mosaic attenuation) or non-inflammatory/fibrotic processes (reticulation/honeycombing, traction bronchiectasis, emphysema).

Blood lymphocyte count is of known prognostic relevance in systemic disorders like SSC or malignancy [29–31]. Our finding that lymphocyte fraction was consistently negatively correlated with the extent of HRCT abnormalities considered “inflammatory” suggests that similar mechanisms may be present in ILD. In BAL, however, lymphocyte fraction showed the opposite behavior with distinctly higher values in the presence of GGO and CON, but also NOD and MOS. Lymphocytosis in BAL is a common finding in inflammatory ILD presenting with patterns of NSIP or OP [5, 32, 33], and especially in ILD associated with formation of granulomas, such as sarcoidosis or hypersensitivity pneumonitis [25]. Conversely, BAL lymphocyte counts are reportedly lower with increasing fibrosis [7, 34].

Similar to our observation of PBL eosinophil fraction being positively correlated with RET and TBR, blood eosinophilia has been reported to be associated with disease severity and presence of ILD in SSC [35]. Elevated eosinophil counts in BAL fluid have repeatedly been described in IPF patients as well as in fibrotic rather than in cellular NSIP [7, 32, 34]. Furthermore, it has been suggested that higher BAL eosinophil counts in IPF patients are associated with an increased risk of acute exacerbations and inferior survival rates [36, 37]. In our patient cohort, such findings for BAL eosinophil fraction could not be shown, possibly due to the low number of patients presenting with significant BAL eosinophilia. The role of eosinophils in the pathogenesis of ILD has not been comprehensively understood yet: cytokines involved in eosinophil activation like Interleukin(IL)-4, IL-5, IL-13 or IL-33 also play a major role in the pathophysiology of IPF and SSCILD [10, 35, 38, 39]. However, it is still unknown, whether an increased eosinophil count in blood or BAL fluid has fundamental pathogenetic implications or rather represents a bystander phenomenon of the molecular processes underlying pulmonary fibrosis. Our findings and the limited existing evidence suggest that an increased eosinophil count may more likely be an indicator of ILD severity and extent than a causative factor.

The close relationship observed between CRP and GGO/CON resembles reports of elevated CRP in ILD patients presenting with patterns like NSIP or OP [5, 7, 15]. In ILD associated with autoimmune disorders, alterations to systemic inflammatory parameters like CRP have been frequently reported. They also pose a risk factor for the development of pulmonary involvement in such conditions [30, 40–42]. Contrary to CRP, we found that lactate dehydrogenase (LDH) was significantly positively correlated with multiple both non-inflammatory/fibrotic and inflammatory HRCT finding scores. Elevation in LDH has been reported in IPF, where it may be associated with functional impairment and may have prognostic properties [43]. Our findings however suggest that LDH could rather be a biomarker reflecting general disease extent and severity than the underlying pathogenetic processes.

Several limitations to our reported study need to be addressed: next to its retrospective, single center approach, the sample size was limited. The reported collective represents a heterogeneous group of several different ILD entities, also including a minority of patients without signs of reticulation or honeycombing (17%). The study collective was derived from patients subsequently discussed by the local ILD-board, which could have increased the likelihood of inclusion of rather complex ILD cases, while typical ILD like for example sarcoidosis may be underrepresented. However, our reported evaluation explicitly did not focus on distinct ILD diagnoses, but on HRCT imaging findings and their association with biomarkers of systemic and local inflammation. Radiological assessment was not accomplished in a blinded fashion but in the presence of the ILD-board, which reflects the multidisciplinary approach to ILD. Our reported scoring system has not been validated in a larger patient cohort but is simple to perform and does not require additional tools like special software. It was not our aim to create a comprehensive HRCT quantification and classification tool but to allow semiquantitative statistical analyses of HRCT scans beyond only “present or absent”. It is obvious that PBL cell counts and biomarkers like CRP or LDH can be substantially altered by infections, neoplastic or hematological conditions. Also, BAL differential cell counts can be influenced by presence of infection, smoking status or age [26, 44]. Additionally, BAL was only performed in approximately half of the patient collective, for it had either been done previously or it was deemed clinically unnecessary. For BAL analyses, this could have led to a selection of patients with uncommon presentation in HRCT or with rather acute than chronic ILD, as suggested by the comparably high mean lymphocyte and neutrophil counts reported. Concerning statistical methods, we acknowledge, that numerous associations have been evaluated for statistical significance, which brings up the issue of multiple testing. Concerning this matter, it was not our aim to test for significance of certain associations, but rather to apply an experimental, hypothesis-building approach aiming to extract clinically relevant biomarkers from a large, diverse dataset. Seeking to overcome these methodological challenges, we primarily used descriptive statistical evaluation like Spearman correlation coefficients and graphical presentation. We chose clinically meaningful quantification categories (no or little, medium, or abundant involvement) for HRCT findings and used the Kruskal–Wallis test for significance testing due to its robustness against outliers.

## Conclusion

We conclude that blood lymphocyte and eosinophil fraction, NLR, CRP and BAL lymphocyte fraction may help to differentiate between non-inflammatory/fibrotic and active inflammatory ILD phenotypes. Especially in ILD with multiple coexisting HRCT abnormalities, these biomarkers could aid the decision whether to primarily initiate anti-inflammatory or antifibrotic treatment. Subsequent prospective and larger-scale trials are warranted to further evaluate the implications of these biomarkers on response to either therapeutic approach.

## Supplementary Information

Below is the link to the electronic supplementary material.

Supplementary file 1 (DOCX 41 kb)

Supplementary file 2 (DOCX 93 kb)

Supplementary file 3 (DOCX 217 kb)

Supplementary file 4 (DOCX 68 kb)

Supplementary file 5 (DOCX 47 kb)

## Data Availability

According to the terms imposed by the ethics committees, the full dataset analyzed during the current study cannot be made publicly available, as it contains possibly identifiable patient data. Upon reasonable request to the corresponding author and if approved as an amendment by the responsible local ethics committee, selected anonymized data can however be shared.
