# Supplementary material for: Computed Tomography Findings as Determinants of Local and Systemic Inflammation Biomarkers in Interstitial Lung Diseases: A Retrospective Registry-Based Descriptive Study
Source: Lung. 2021 Mar 26;199(2):155–64. doi: 10.1007/s00408-021-00434-w (PMC8053160; doi:10.1007/s00408-021-00434-w)
Supplement: Supplementary file 1 — (DOCX 41 kb) [file 408_2021_434_MOESM1_ESM.docx]

| 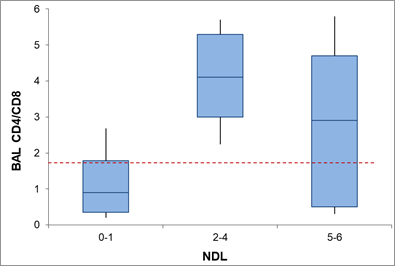 |
| --- |

Supplementary figure 1. Boxplot diagram of BAL CD4+/CD8+ ratio according to NDL score categories. Boxplot diagrams depict the median (line), the upper and lower quartile (boxes) and the 95% confidence intervals (whiskers). The red dashed line represents the median of all values. BAL=broncho-alveolar lavage, CD=cluster of differentiation, NDL=parenchymal nodules
