# Supplementary material for: Computed Tomography Findings as Determinants of Local and Systemic Inflammation Biomarkers in Interstitial Lung Diseases: A Retrospective Registry-Based Descriptive Study
Source: Lung. 2021 Mar 26;199(2):155–64. doi: 10.1007/s00408-021-00434-w (PMC8053160; doi:10.1007/s00408-021-00434-w)
Supplement: Supplementary file 2 — (DOCX 93 kb) [file 408_2021_434_MOESM2_ESM.docx]

| **HRCT finding scores (median, range)** | **IPAF** | **IPF** | **CHP** | **iNSIP** | **aILD** | **Unclassified ILD** | **Other ILD** |
| --- | --- | --- | --- | --- | --- | --- | --- |
| RET | 5 (0-6) | 6 (2-6) | 6 (0-6) | 6 (2-6) | 4 (2-6) | 4 (0-6) | 0 (0-6) |
| TBR | 2 (0-6) | 4 (0-6) | 4 (0-6) | 3 (0-6) | 2 (0-5) | 3 (0-6) | 1 (0-4) |
| EMP | 0 (0-2) | 0 (0-6) | 0 (0-2) | 0 (0-2) | 0 (0-0) | 0 (0-0) | 0 (0-4) |
| GGO | 0 (0-6) | 0 (0-6) | 2 (0-6) | 0 (0-6) | 0 (0-6) | 0 (0-6) | 0 (0-6) |
| NDL | 0 (0-6) | 0 (0-0) | 2 (0-6) | 0 (0-6) | 0 (0-6) | 0 (0-6) | 1 (0-6) |
| CON | 0.5 (0-6) | 0 (0-4) | 0 (0-2) | 0 (0-6) | 0 (0-3) | 0 (0-6) | 1 (0-6) |
| MOS | 0 (0-6) | 0 (0-2) | 3 (0-6) | 0 (0-6) | 0 (0-3) | 0 (0-4) | 0 (0-6) |
| **Peripheral blood  biomarkers (mean, SD)** | **IPAF** | **IPF** | **CHP** | **iNSIP** | **aILD** | **Unclassified ILD** | **Other ILD** |
| PBL LEU (G/L) | 8.6 (3.0) | 8.4 (2.5) | 8.2 (2.3) | 9.4 (3.3) | 7.5 (3.7) | 8.2 (3.1) | 10.0 (5.0) |
| PBL NEU (%) | 74.4 (10.2) | 66.2 (10.7) | 70.6 (7.5) | 70.9 (11.4) | 73.7 (10.8) | 69.9 (11.0) | 70.8 (13.5) |
| PBL LYM (%) | 18.2 (8.4) | 24.9 (8.6) | 20.3 (6.6) | 19.6 (8.6) | 19.3 (8.0) | 19.9 (8.3) | 20.5 (10.7) |
| PBL NLR | 6.2 (5.9) | 3.4 (2.9) | 4.0 (1.7) | 4.8 (3.3) | 4.8 (2.9) | 4.7 (3.5) | 6.4 (8.1) |
| PBL EOS (%) | 1.4 (1.5) | 2.5 (2.3) | 2.3 (1.9) | 2.9 (4.0) | 2.0 (2.5) | 3.9 (4.1) | 2.0 (2.5) |
| PBL CRP (mg/dL) | 2.1 (3.1) | 1.4 (2.2) | 1.3 (1.8) | 0.7 (1.0) | 0.6 (0.5) | 2.8 (4.6) | 1.7 (2.2) |
| PBL LDH (U/L) | 243 (56.2) | 230.8 (49.1) | 273 .4 (101.3) | 259.6 (85.7) | 302.7 (126.1) | 257.7 (77.8) | 206.0 (51.9) |
| **BAL biomarkers  (mean, SD)** | **IPAF** | **IPF** | **CHP** | **iNSIP** | **aILD** | **Unclassified ILD** | **Other ILD** |
| BAL MAK (%) | 44.7 (28.2) | 60.1 (31.9) | 30.0 (21.0) | 81.9 (10.2) | 61.3 (39.0) | 34.7 (16.3) | 48.9 (31.0) |
| BAL NEU (%) | 21.8 (23.5) | 29.3 (30.5) | 30.9 (34.0) | 6.9 (7.7) | 4.5 (4.2) | 24.8 (23.9) | 12.8 (13.4) |
| BAL EOS (%) | 4.1 (3.9) | 7.6 (8.2) | 12.0 (19.4) | 1.5 (1.3) | 2.0 (2.8) | 12.0 (7.0) | 3.8 (1.5) |
| BAL LYM (%) | 24.8 (27.1) | 6.5 (6.3) | 24.1 (24.8) | 10.0 (8.2) | 18 (2-89) | 18.4 (9.9) | 28.1 (23.1) |

Supplementary table 1. HRCT findings, peripheral blood and bronchoalveolar lavage characteristics according to ILD-board diagnoses. Values are given as median (range) or mean (SD) as specified. SD=standard deviation, HRCT=high-resolution computed tomography, ILD=interstitial lung disease, IPAF=interstitial pneumonia with autoimmune features, IPF=idiopathic pulmonary fibrosis, CHP=chronic hypersensitivity pneumonitis, iNSIP=idiopathic non-specific interstitial pneumonia, aILD=autoimmune-associated ILD, RET=reticulation/honeycombing, TBR=traction bronchiectasis, EMP=emphysema, GGO=ground glass opacities, CON=consolidations, NDL=parenchymal nodules, MOS=mosaic attenuation, PBL=peripheral blood, LEU= leukocyte count, NEU=neutrophil fraction, LYM=lymphocyte fraction, NLR= neutrophil to lymphocyte ratio, EOS=eosinophil fraction, CRP=C-reactive protein, LDH=lactate dehydrogenase, BAL=broncho-alveolar lavage, MAK=macrophage fraction
