# Supplementary material for: Computed Tomography Findings as Determinants of Local and Systemic Inflammation Biomarkers in Interstitial Lung Diseases: A Retrospective Registry-Based Descriptive Study
Source: Lung. 2021 Mar 26;199(2):155–64. doi: 10.1007/s00408-021-00434-w (PMC8053160; doi:10.1007/s00408-021-00434-w)
Supplement: Supplementary file 3 — (DOCX 217 kb) [file 408_2021_434_MOESM3_ESM.docx]

|  | **Median in HRCT score groups (range)** | | | **p** | **n** | | |
| --- | --- | --- | --- | --- | --- | --- | --- |
| **PBL LEU (G/L)** | **0 - 1** | **2 - 4** | **5 – 6** |  | **0 - 1** | **2 - 4** | **5 - 6** |
| RET | 7.3 (4.8-15.9) | 7.5 (3.8-25.5) | 8.5 (4.5-16.1) | 0.617 | 22 | 46 | 54 |
| TBR | 7.8 (4.6-25.5) | 7.5 (3.8-19.1) | 8.5 (4.8-14.6) | 0.537 | 30 | 66 | 26 |
| EMP | 7.8 (3.8-19.1) | 8.6 (4.6-25.5) | 7.2 (4.8-14.6) | 0.916 | 100 | 19 | 3 |
| GGO | 7.6 (3.8-16.1) | 7.9 (4.2-14.6) | 8.3 (4.8-25.5) | 0.449 | 78 | 24 | 20 |
| CON | 7.8 (3.8-25.5) | 7.4 (4.5-19.1) | 9.7 (4.8-14.6) | 0.811 | 93 | 21 | 8 |
| NDL | 7.6 (3.8-19.1) | 8.3 (4.2-25.5) | 9.6 (5.6-15.4) | 0.117 | 85 | 24 | 13 |
| MOS | 7.7 (3.8-25.5) | 8.4 (4.2-13.3) | 7.6 (4.8-18.1) | 0.839 | 96 | 18 | 8 |
| **PBL NEU (%)** | **0 - 1** | **2 - 4** | **5 – 6** | **p** | **0 - 1** | **2 - 4** | **5 - 6** |
| RET | 74.5 (54.4-91.7) | 72.4 (46.2-96.6) | 68.8 (47.6-88.3) | 0.362 | 22 | 54 | 54 |
| TBR | 74.3 (46.2-94.9) | 69.0 (46.2-94.9) | 73.1 (47.6-86.3) | 0.130 | 30 | 65 | 26 |
| EMP | 71.3 (51.4-94.9) | 69.6 (46.2-96.6) | 69.8 (47.6-86.3) | 0.967 | 99 | 19 | 3 |
| GGO | 69.7 (46.2-91.7) | 71.9 (51.9-89.6) | 74.8 (47.6-96.6) | 0.289 | 78 | 24 | 19 |
| CON | 69.8 (46.2-96.6) | 71.5 (51.4-86.3) | 79.5 (58.2-89.6) | 0.077 | 93 | 20 | 8 |
| NDL | 69.5 (46.2-89.6) | 72.8 (54.4-96.6) | 71.4 (51.9-91.7) | 0.217 | 84 | 24 | 13 |
| MOS | 69.6 (46.2-96.6) | 72.6 (51.4-90.2) | 79.3 (61.0-94.9) | 0.108 | 95 | 18 | 8 |
| **PBL LYM (%)** | **0 - 1** | **2 - 4** | **5 – 6** | **p** | **0 - 1** | **2 - 4** | **5 - 6** |
| RET | 19.3 (4.6-37.4) | 19.1 (2.6-41.9) | 22.3 (4.6-38.2) | 0.366 | 22 | 45 | 54 |
| TBR | 18.6 (2.6-37.4) | 21.4 (3.4-41.9) | 19.3 (5.9-38.2) | 0.337 | 30 | 65 | 26 |
| EMP | 20.0 (3.4-38.0) | 20.6 (2.6-41.9) | 23.2 (5.9-38.2) | 0.915 | 99 | 19 | 3 |
| GGO | **22.7 (4.6-41.9)** | **16.9 (7.8-41.5)** | **17.9 (2.6-38.2)** | **0.032** | 78 | 24 | 19 |
| CON | **21.0 (2.6-41.9)** | **19.7 (5.9-34.2)** | **13.0 (7.8-21.5)** | **0.027** | 93 | 20 | 8 |
| NDL | 21.1 (4.6-41.9) | 18.4 (2.6-37.4) | 21.8 (4.6-41.5) | 0.150 | 84 | 24 | 13 |
| MOS | 20.9 (2.6-41.9) | 18.2 (6.6-34.8) | 15.6 (3.4-24.4) | 0.154 | 95 | 18 | 8 |
| **PBL NLR** | **0 - 1** | **2 - 4** | **5 – 6** | **p** | **0 - 1** | **2 - 4** | **5 - 6** |
| RET | 4.2 (1.5-19.9) | 3.8 (1.1-37.1) | 3.1 (1.2 - 19.3) | 0.311 | 22 | 45 | 54 |
| TBR | 4.1 (1.5 -37.1) | 3.2 (1.1-27.8) | 3.9 (1.2-14.7) | 0.160 | 30 | 65 | 26 |
| EMP | 3.7 (1.4 -27.8) | 3.5 (1.1-37.1) | 3.0 (1.2-14.7) | 0.941 | 99 | 19 | 3 |
| GGO | 3.2 (1.1-19.9) | 4.2 (1.3-11.4) | 4.2 (1.2-37.1) | 0.095 | 78 | 24 | 19 |
| CON | **3.2 (1.1-37.1)** | **3.7 (1.5-14.7)** | **6.4 (5.1-11.4)** | **0.009** | 93 | 20 | 8 |
| NDL | 3.5 (1.1-19.3) | 4.0 (1.5-37.1) | 3.2 (1.3-19.9) | 0.172 | 84 | 24 | 13 |
| MOS | 3.4 (1.1-37.1) | 4.1 (1.5-13.3) | 5.1 (2.5-27.8) | 0.156 | 95 | 18 | 8 |
| **PBL EOS (%)** | **0 - 1** | **2 - 4** | **5 – 6** | **p** | **0 - 1** | **2 - 4** | **5 - 6** |
| RET | 1.0 (0.0-5.1) | 1.4 (0.0-13.8) | 1.8 (0.1-14.7) | 0.092 | 21 | 45 | 52 |
| TBR | **0.9 (0.0-4.4)** | **1.7 (0.0-13.8)** | **1.9 (0.1-14.7)** | **0.006** | 30 | 64 | 24 |
| EMP | 1.5 (0.0-14.7) | 1.5 (0.1-13.8) | 1.8 (0.1-6.5) | 0.909 | 96 | 19 | 3 |
| GGO | 1.5 (0.0-9.5) | 1.5 (0.0-14.7) | 1.4 (0.0-11.2) | 0.973 | 75 | 24 | 19 |
| CON | 1.5 (0.0-13.8) | 1.7 (0.1-14.7) | 0.6 (0.1-2.5) | 0.168 | 91 | 19 | 8 |
| NDL | 1.5 (0.0-14.7) | 1.6 (0.0-13.8) | 1.1 (0.0-11.2) | 0.753 | 82 | 23 | 13 |
| MOS | 1.5 (0.0-14.7) | 1.8 (0.0-8.4) | 0.3 (0.0-8.6) | 0.148 | 93 | 17 | 8 |
| **PBL CRP (mg/dL)** | **0 - 1** | **2 - 4** | **5 – 6** | **p** | **0 - 1** | **2 - 4** | **5 - 6** |
| RET | 0.5 (0.1-7.1) | 0.4 (0.1-8.4) | 0.9 (0.1-15.9) | 0.257 | 22 | 45 | 56 |
| TBR | 0.4 (0.1-7.1) | 0.7 (0.1-12.3) | 0.3 (0.1-15.9) | 0.332 | 30 | 67 | 26 |
| EMP | 0.5 (0.1-15.9) | 0.6 (0.1-12.3) | 0.3 (0.1-5.0) | 0.917 | 101 | 19 | 3 |
| GGO | **0.4 (0.1-12.3)** | **1.0 (0.1-15.9)** | **0.9 (0.1-8.4)** | **0.023** | 80 | 23 | 20 |
| CON | **0.5 (0.1-12.3)** | **0.4 (0.1-15.9)** | **4.1 (0.6-8.4)** | **0.004** | 93 | 22 | 8 |
| NDL | 0.4 (0.1-15.9) | 0.9 (0.1-6.8) | 0.7 (0.1-7.1) | 0.221 | 87 | 23 | 13 |
| MOS | 0.5 (0.1-15.9) | 0.7 (0.1-3.8) | 0.9 (0.2-6.0) | 0.393 | 98 | 17 | 8 |
| **PBL LDH (U/L)** | **0 - 1** | **2 - 4** | **5 – 6** | **p** | **0 - 1** | **2 - 4** | **5 - 6** |
| RET | **199 (131-372)** | **236 (113-614)** | **244 (152-426)** | **0.010** | 20 | 42 | 51 |
| TBR | **195 (125-394)** | **245 (113-614)** | **259 (188-587)** | **<0.001** | 26 | 61 | 26 |
| EMP | 236 (113-614) | 200 (152-306) | 232 (181-340) | 0.210 | 94 | 16 | 3 |
| GGO | **224 (113-397)** | **244 (153-614)** | **261 (194-587)** | **0.049** | 74 | 21 | 18 |
| CON | 233 (113-614) | 228 (131-390) | 293 (211-426) | 0.115 | 88 | 19 | 6 |
| NDL | 232 (113-397) | 231 (153-587) | 266 (171-614) | 0.196 | 90 | 24 | 13 |
| MOS | **226 (113-614)** | **267 (167-354)** | **259 (220-587)** | **0.027** | 89 | 16 | 8 |

Supplementary table 2. Peripheral blood biomarkers according to HRCT finding categories. Data are given as median (range). The p-value is for statistical significance of differences (p<0.05) between the groups was calculated using the Kruskal-Wallis test. Significant association are shown in bold letters and blue color for positive, red for negative associations. HRCT=high-resolution computed tomography, PBL=peripheral blood, LEU=leukocyte count, NEU=neutrophil fraction, LYM=lymphocyte fraction, NLR=neutrophil to lymphocyte ratio, EOS= eosinophil fraction, CRP=C-reactive protein, LDH=lactate dehydrogenase, RET=reticulation/honeycombing, TBR=traction bronchiectasis, EMP=emphysema, GGO=ground glass opacities, CON=consolidations, NDL=parenchymal nodules, MOS=mosaic attenuation
