# Supplementary material for: Computed Tomography Findings as Determinants of Local and Systemic Inflammation Biomarkers in Interstitial Lung Diseases: A Retrospective Registry-Based Descriptive Study
Source: Lung. 2021 Mar 26;199(2):155–64. doi: 10.1007/s00408-021-00434-w (PMC8053160; doi:10.1007/s00408-021-00434-w)
Supplement: Supplementary file 4 — (DOCX 68 kb) [file 408_2021_434_MOESM4_ESM.docx]

|  | **Median in HRCT score groups (range)** | | | **p** | **n** | | |
| --- | --- | --- | --- | --- | --- | --- | --- |
| **BAL MAK (%)** | **0 - 1** | **2 - 4** | **5 - 6** |  | **0 - 1** | **2 - 4** | **5 - 6** |
| RET | 37.0 (6.0-95.0) | 49.5 (5.0-98.0) | 62.0 (5.0-93.0) | 0.694 | 13 | 18 | 35 |
| TBR | 25.0 (5.0-98.0) | 61.0 (5.0-98.0) | 67.0 (7.0-90.0) | 0.327 | 17 | 35 | 14 |
| EMP | 61.0 (5.0-98.0) | 48.5 (7.0-84.0) | 7.0 (7.0-7.0) | 0.308 | 55 | 10 | 1 |
| GGO | 57.0 (5.0-98.0) | 52.5 (5.0-82.0) | 34.5 (7.0-87.0) | 0.565 | 46 | 10 | 10 |
| CON | 53.0 (5.0-98.0) | 46.0 (6.0-90.0) | 69.0 (19.0-87.0) | 0.908 | 50 | 13 | 3 |
| NDL | 63.0 (6.0-98.0) | 46.5 (17.0-95.0) | 37.0 (5.0-85.0) | 0.405 | 42 | 14 | 10 |
| MOS | 61.5 (5.0-98.0) | 53.0 (23.0-93.0) | 20.0 (5.0-66.0) | 0.092 | 54 | 7 | 5 |
| **BAL NEU (%)** | **0 - 1** | **2 - 4** | **5 - 6** | **p** | **0 - 1** | **2 - 4** | **5 - 6** |
| RET | 12.0 (1.0-65.0) | 7.5 (0.0-89.0) | 10.0 (0.0-84.0) | 0.520 | 13 | 16 | 33 |
| TBR | 11.0 (0.0-89.0) | 7.5 (0.0-84.0) | 13.0 (1.0-64.0) | 0.691 | 16 | 32 | 14 |
| EMP | 10.0 (0.0-89.0) | 8.0 (0.0-66.0) | 58.0 (58.0-58.0) | 0.452 | 52 | 9 | 1 |
| GGO | 10.0 (0.0-89.0) | 6.5 (0.0-24.0) | 9.5 (1.0-61.0) | 0.209 | 42 | 10 | 10 |
| CON | 9.5 (0.0-89.0) | 10.0 (2.0-61.0) | 7.0 (3.0-19.0) | 0.873 | 46 | 13 | 3 |
| NDL | 10.0 (0.0-89.0) | 10.5 (1.0-70.0) | 10.0 (0.0-84.0) | 0.914 | 39 | 14 | 9 |
| MOS | 9.0 (0.0-89.0) | 10.0 (4.0-70.0) | 12.0 (1.0-84.0) | 0.739 | 50 | 7 | 5 |
| **BAL EOS (%)** | **0 - 1** | **2 - 4** | **5 - 6** | **p** | **0 - 1** | **2 - 4** | **5 - 6** |
| RET | 3.0 (2.0-4.0) | 4.0 (0.0-23.0) | 4.0 (0.0-55.0) | 0.876 | 4 | 11 | 21 |
| TBR | 3.5 (0.0-4.0) | 3.0 (0.0-55.0) | 5.0 (0.0-19.0) | 0.699 | 6 | 21 | 9 |
| EMP | 3.0 (0.0-55.0) | 6.0 (0.0-23.0) | - | 0.547 | 29 | 7 | - |
| GGO | 3.0 (0.0-23.0) | 4.0 (0.0-55.0) | 5.0 (1.0-10.0) | 0.891 | 26 | 5 | 5 |
| CON | 3.5 (0.0-55.0) | 3.0 (1.0-19.0) | 4.0 (4.0-4.0) | 0.978 | 28 | 7 | 1 |
| NDL | 3.0 (0.0-55.0) | 3.0 (0.0-13.0) | 4.5 (0.0-12.0) | 0.835 | 21 | 9 | 6 |
| MOS | 3.0 (0.0-55.0) | 7.0 (0.0-13.0) | 5.0 (3.0-6.0) | 0.794 | 29 | 4 | 3 |
| **BAL LYM (%)** | **0 - 1** | **2 - 4** | **5 - 6** | **p** | **0 - 1** | **2 - 4** | **5 - 6** |
| RET | 15.0 (2.0-68.0) | 13.0 (0.0-89.0) | 11.0 (0.0-88.0) | 0.578 | 12 | 17 | 34 |
| TBR | 11.0 (0.0-88.0) | 13.0 (0.0-89.0) | 11.5 (4.0-74.0) | 0.921 | 15 | 34 | 14 |
| EMP | 14.5 (1.0-89.0) | 7.5 (0.0-43.0) | 4.0 (4.0-4.0) | 0.249 | 52 | 10 | 1 |
| GGO | **7.5 (0.0-77.0)** | **21.0 (7.0-89.0)** | **17.0 (4.0-88.0)** | **0.017** | 44 | 10 | 9 |
| CON | 9.5 (0.0-89.0) | 17.5 (2.0-77.0) | 24.0 (16.0-54.0) | 0.783 | 48 | 12 | 3 |
| NDL | 9.0 (0.0-88.0) | 16.5 (2.0-74.0) | 23.0 (2.0-89.0) | 0.344 | 41 | 14 | 8 |
| MOS | 11.0 (0.0-89.0) | 15.0 (3.0-33.0) | 33.0 (2.0-74.0) | 0.223 | 51 | 7 | 5 |

Supplementary table 3. Broncho-alveolar lavage biomarkers according to HRCT finding score categories. Data are given as median (range). The p-value for statistical significance of differences (p<0.05) between the groups was calculated using the Kruskal-Wallis test. Significant association are shown in bold letters and blue color for positive associations.
HRCT=high-resolution computed tomography, BAL=broncho-alveolar lavage, MAK=macrophage fraction, NEU=neutrophil fraction, EOS=eosinophil fraction, LYM=lymphocyte fraction, RET=reticulation/honeycombing, TBR=traction bronchiectasis, EMP=emphysema, GGO=ground glass opacities, CON=consolidations, NDL=parenchymal nodules, MOS=mosaic attenuation
