# Supplementary material for: Computed Tomography Findings as Determinants of Local and Systemic Inflammation Biomarkers in Interstitial Lung Diseases: A Retrospective Registry-Based Descriptive Study
Source: Lung. 2021 Mar 26;199(2):155–64. doi: 10.1007/s00408-021-00434-w (PMC8053160; doi:10.1007/s00408-021-00434-w)
Supplement: Supplementary file 5 — (DOCX 47 kb) [file 408_2021_434_MOESM5_ESM.docx]

| **CD4/CD8** | **0 - 1** | **2 - 4** | **5 - 6** | **p** | **0 - 1** | **2 - 4** | **5 - 6** |
| --- | --- | --- | --- | --- | --- | --- | --- |
| RET | 1.8 (0.5-4.1) | 0.9 (0.3-5.3) | 2.1 (0.2-5.8) | 0.804 | 5 | 5 | 10 |
| TBR | 0.9 (0.3-3.0) | 1.9 (0.2-5.8) | 4.7 (4.1-5.3) | 0.088 | 6 | 11 | 3 |
| EMP | 1.4 (0.2-5.8) | 4.2 (2.7-5.7) |  | 0.166 | 18 | 2 | 0 |
| GGO | 1.9 (0.4-4.7) | 0.5 (0.2-5.8) | 4.7 (0.3-5.7) | 0.325 | 11 | 5 | 4 |
| CON | 2.2 (0.3-5.3) | 0.9 (0.2-5.7) | 3.4 (1.0-5.8) | 0.428 | 13 | 5 | 2 |
| NDL | **0.9 (0.2-2.7)** | **4.1 (2.2-5.7)** | **2.9 (0.3-5.8)** | **0.017** | 10 | 5 | 5 |
| MOS | 1.0 (0.2-5.8) | 2.2 (0.9-2.9) | 3.0 (0.5-5.3) | 0.684 | 13 | 3 | 4 |

Supplementary table 4. CD4+/CD8+ ratio from broncho-alveolar lavage according to HRCT finding categories. Data are given as median (range). The p-value for statistical significance of differences (p<0.05) between the groups was calculated using the Kruskal-Wallis test. Significant association are shown in bold letters and blue color for positive associations. RET=reticulation/honeycombing, TBR=traction bronchiectasis, EMP=emphysema, GGO=ground glass opacities, CON=consolidations, NDL=parenchymal nodules, MOS=mosaic attenuation, CD=cluster or differentiation, HRCT=high-resolution computed tomography
